# DOHAD and early pubertal timing

**DOI:** 10.1186/1687-9856-2015-S1-O12

**Published:** 2015-04-28

**Authors:** Ken K Ong

**Affiliations:** 1 MRC Epidemiology Unit, Institute of Metabolic Science, University of Cambridge, Cambridge, UK

## 

Age at menarche varies widely between girls, is estimated to be highly heritable and is associated with long-term health outcomes, such as obesity and type 2 diabetes. Earlier pubertal maturation in boys and girls links rapid postnatal growth weight gain to later life metabolic and cardiovascular disease [1,2]. Genome-wide association studies (GWAS), which genotype hundreds of thousands of common genetic variants located across the entire genome, have been successful in identifying many specific genetic determinants of pubertal timing and these findings have informed the mechanisms that link earlier pubertal timing to increased risks of disease. The genetic signals indicate that both obesity-related and obesity-independent mechanisms may link pubertal timing to insulin sensitivity and diabetes risk.

Our earlier findings [3] in the ReproGen international GWAS consortium identified substantial overlap between loci associated with menarche and those associated with body mass index, as had been predicted by analyses of data from twins, and in keeping with recognised associations between infancy and childhood weight gain and pubertal timing, and in turn between pubertal timing and obesity in adult life. However, the locus with the strongest individual effect size is in the gene LIN28B, which regulates microRNA processing and also insulin sensitivity. Our recent larger studies indicate further mechanisms that may link puberty timing to diabetes risk, and also suggest pubertal timing as a possible postnatal development target for the evolution of genomic imprinting [4].
